# Role of Sphingomyelinase in Infectious Diseases Caused by *Bacillus cereus*

**DOI:** 10.1371/journal.pone.0038054

**Published:** 2012-06-06

**Authors:** Masataka Oda, Manabu Hashimoto, Masaya Takahashi, Yuka Ohmae, Soshi Seike, Ryoko Kato, Aoi Fujita, Hideaki Tsuge, Masahiro Nagahama, Sadayuki Ochi, Teppei Sasahara, Shunji Hayashi, Yoshikazu Hirai, Jun Sakurai

**Affiliations:** 1 Department of Microbiology, Faculty of Pharmaceutical Sciences, Tokushima Bunri University, Yamashiro-cho, Tokushima, Japan; 2 Institute for Health Sciences, Tokushima Bunri University, Yamashiro-cho, Tokushima, Japan; 3 Faculty of Life Sciences, Kyoto Sangyo University, Kamigamo Motoyama Kita-ku, Kyoto, Japan; 4 School of Medicine, Fujita Health University, Toyoake, Aichi, Japan; 5 School of Medicine, Jichi Medical University, Shimono-city, Tochigi, Japan; The Scripps Research Institute, United States of America

## Abstract

*Bacillus cereus* (*B. cereus*) is a pathogen in opportunistic infections. Here we show that *Bacillus cereus* sphingomyelinase (*Bc*-SMase) is a virulence factor for septicemia. Clinical isolates produced large amounts of *Bc*-SMase, grew in vivo, and caused death among mice, but ATCC strains isolated from soil did not. A transformant of the ATCC strain carrying a recombinant plasmid containing the *Bc-*SMase gene grew in vivo, but that with the gene for E53A, which has little enzymatic activity, did not. Administration of an anti-*Bc*-SMase antibody and immunization against *Bc*-SMase prevented death caused by the clinical isolates, showing that *Bc*-SMase plays an important role in the diseases caused by *B. cereus*. Treatment of mouse macrophages with *Bc*-SMase resulted in a reduction in the generation of H_2_O_2_ and phagocytosis of macrophages induced by peptidoglycan (PGN), but no effect on the release of TNF-α and little release of LDH under our experimental conditions. Confocal laser microscopy showed that the treatment of mouse macrophages with *Bc*-SMase resulted in the formation of ceramide-rich domains. A photobleaching analysis suggested that the cells treated with *Bc*-SMase exhibited a reduction in membrane fluidity. The results suggest that *Bc*-SMase is essential for the hydrolysis of SM in membranes, leading to a reduction in phagocytosis.

## Introduction

*B. cereus* is well-known for its role as a mediator of food-borne illness [1], [2], [3], [4]. The microorganism, which forms spores, is found worldwide in dust, air, and water [5]. Therefore, *B. cereus* is ubiquitous in the hospital environment, indicating that contamination of dressings, intravenous catheters, and linen provides an opportunity for infection [5]. It is possible that *B. cereus* is a pathogen of nosocomial infections transmitted via towels, linen, and balloons to compromised patients [6], [7]. In recent years, there has been an increasing appreciation for its potential as an opportunistic pathogen in immunocompromised hosts [5], [6], [8], [9]. The microorganism secretes a wide variety of membrane-damaging toxins, phospholipases such as *Bc*-SMase, phosphatidylinositol-specific phospholipase C (PIPLC) and phosphatidylcholine-specific phospholipase C (PCPLC), and hemolysins such as cereolysin O, hemolysins and proteases [3], [10], [11], [12], [13], [14]. However, there has been little research into the contributions of these enzymes and toxins to the infectious diseases caused by *B*. *cereus*.

The SMase produced by the intracellular pathogen *Listeria ivanovii* was shown to mediate bacterial escape from the phagocytic vacuole following internalization, thereby promoting intracellular survival and propagation [15]. *Helicobacter pylori*-derived SMase was found to contribute toward cytotoxicity for gastric cells [16]. β-Hemolysin containing SMase activity from methicillin-resistant *Staphylococcus aureus* was expressed by 91% of strains in a high-toxicity group [17]. A mutant strain with deletions of β-hemolysin and catalase was significantly less virulent to mice than the wild-type *Staphylococcus aureus* strain [18]. We reported that *Bc*-SMase lysed sheep erythrocytes containing large amounts of SM in the outer lipid layer of their plasma membranes [19]. However, the enzyme is known not to be lethal or cytotoxic. *Bc*-SMase belongs to a family of Mg^2+^-dependent neutral SMases (nSMase) that includes SMases produced by *Staphylococcus aureus*, and *Listeria ivanovii* [20]. The members of this family share a high degree of homology in amino acid sequence [20], [21], [22]. However, the role of *Bc*-SMase in the virulence of *B. cereus* remains controversial.

To investigate the relationship between *Bc*-SMase and *B. cereus* infections, we examined the relationship between *Bc*-SMase and the growth in vivo of clinical isolates of *B. cereus*.

## Results

### Pathogenicity of the Clinical Isolates of *B. cereus*

**Figure 1:**
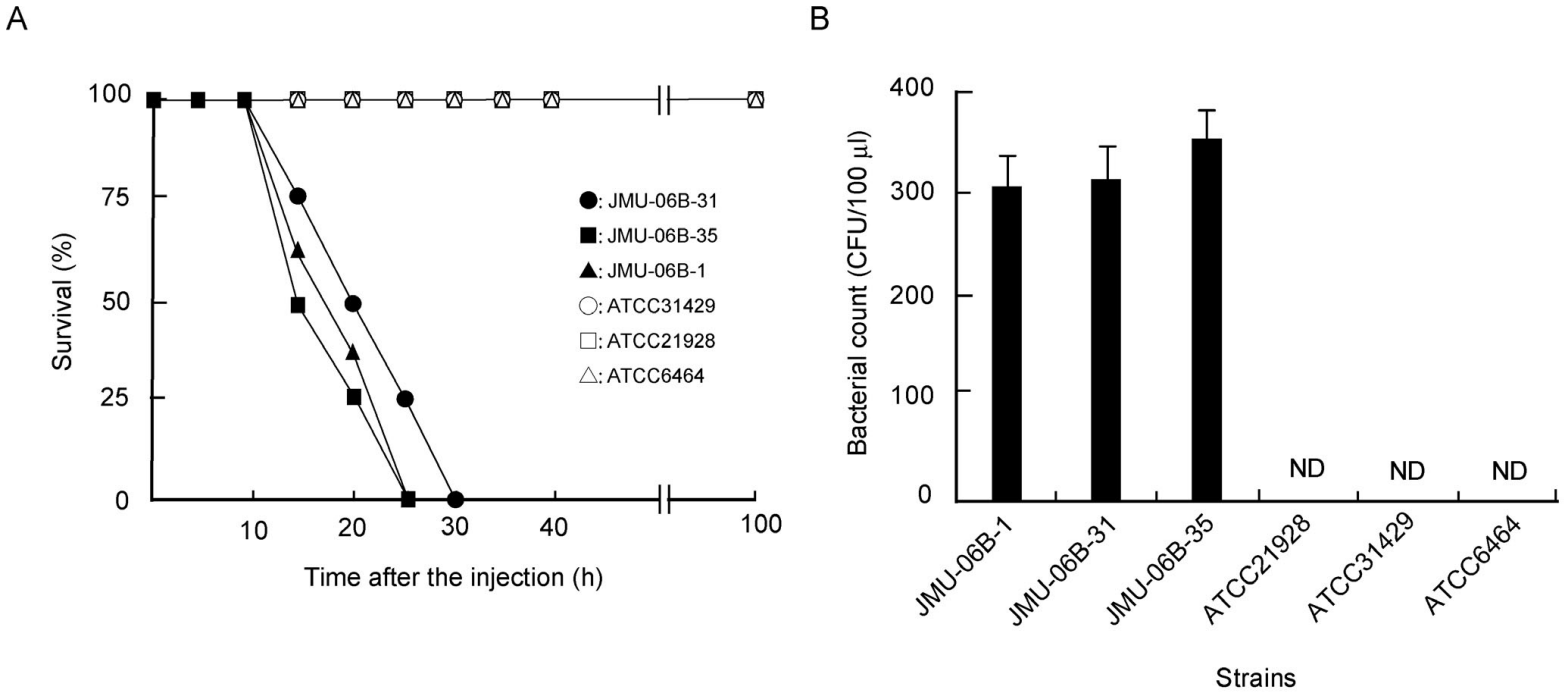
Lethal challenges with clinical isolates and ATCC strains of *B. cereus.* Mice were intraperitoneally administered with clinical isolates and ATCC strains of *B. cereus* (3×10^8^ CFU/mouse). Clinical isolates; JMU-06B-31 (•), JMU-06B-35 (▪), and JMU-06B-1 (▴). ATCC strains*;* ATCC21928 (□), ATCC31429 (○), and ATCC6464 (△). A) Mice were monitored every five hours after the injection. The duration of the experiment was set at 100 h. B) The microorganisms in the blood of mice about 12 h after the administration of various strains were cultured on Luria Broth agar plates. Values represent the mean ± SEM; *n* = 5 independent experiments. ND: not detected.

To investigate if *B. cereus* JMU-06B-31 and JMU-06B-1, isolated from a patient with septicemia, and JMU-06B-35, isolated from a patient with endophthalmitis, grow in mice in vivo, six- to eight-week old male wild-type mice of the ICR mice were each injected intraperitoneally with 5×10^8^ CFU of the clinical isolates or ATCC21928, ATCC31429, and ATCC6464 isolated from soil. Mice administered with the clinical isolates began to die after 12 h, and all mice died within 30 h of the administration (Fig. 1A). Mice injected with ATCC21928, ATCC31429, and ATCC6464 did not die within 100 h (Fig. 1A). The number of microorganisms in the blood of mice about 12 h after the administration of JMU-06B-31, JMU-06B-35, and JMU-06B-1 was 300–400 CFU/100 µL, whereas the ATCC strains were not detected in blood (Fig. 1B).

### Production of Phospholipases by the Clinical Isolates and the ATCC Strains of *B. cereus*

**Figure 2:**
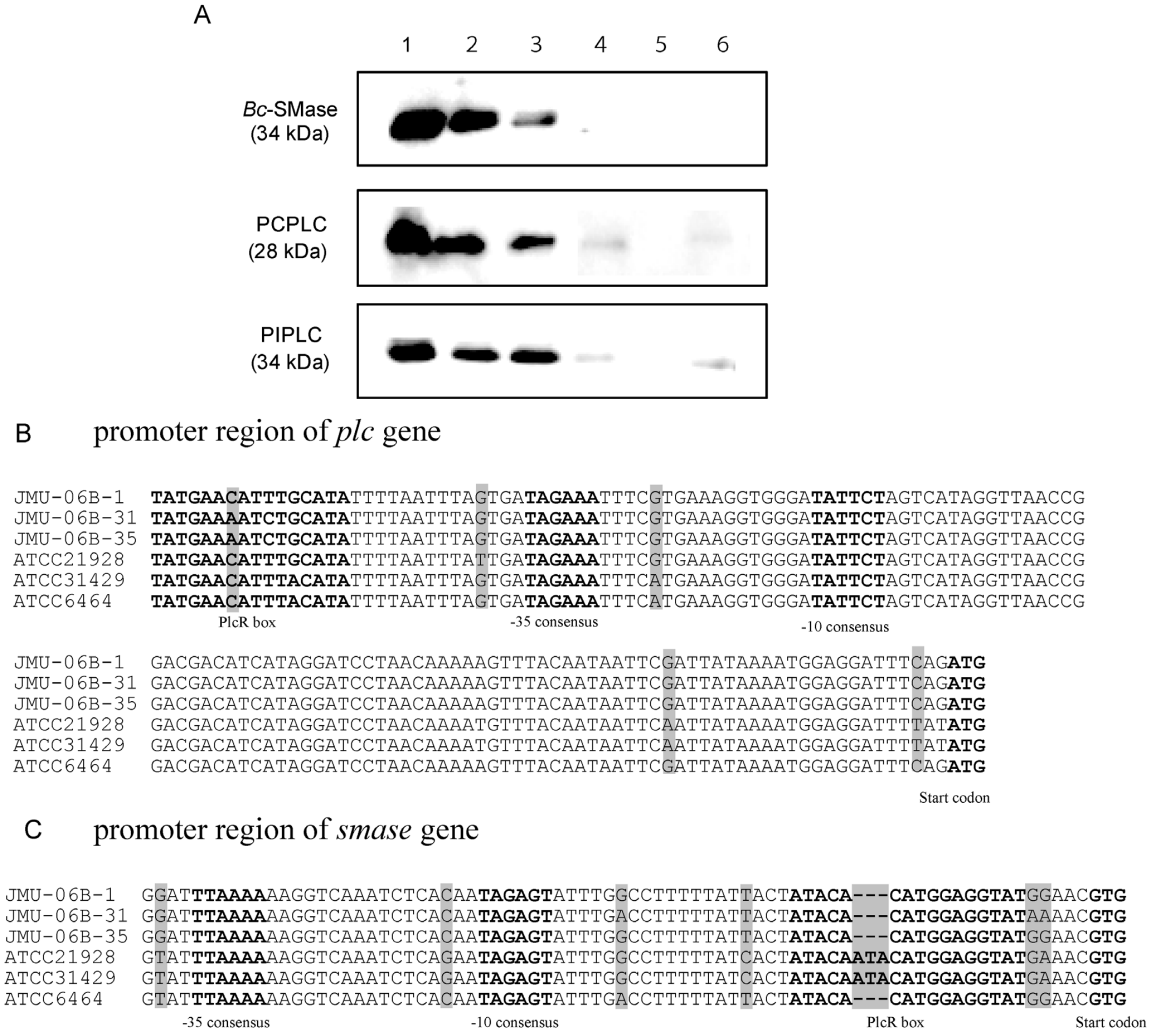
Expression of phospholipases and promoter sequences of *smase* from clinical isolates and ATCC strains of *B. cereus*. A) 50% Ammonium sulfate precipitation fractions of the culture supernatants (1.0 mg protein) were subjected to SDS-PAGE and Western blotting using anti-*Bc*-SMase, -PCPLC, and -PIPLC antibodies. Lane: 1, JMU-06B-31; 2, JMU-06B-35; 3, JMU-06B-1; 4, ATCC21928; 5, ATCC31429; 6, ATCC6464. A representative result from one of three experiments is shown. B, C) The sequences of the promoter region of *plc* and *smase* from clinical isolates and ATCC strains of *B. cereus* were aligned by the program T-Coffee [44]. Consensus sequences of regulatory elements are indicated in bold type. Gray areas indicate nucleotide sequence differences.

Phospholipases produced by bacteria such as *Staphylococcus aureus, Clostridium perfringens,* and *Helicobacter pylori* are reported to be associated with local infections and of importance in the establishment of systemic diseases [4], [16], [23], [24]. To analyze the production of phospholipases by *B. cereus*, we measured the amount of phospholipases produced by the clinical isolates and the ATCC strains in Luria Broth medium. These strains were cultured to an optical density at 620 nm of 0.8 in the medium. The enzyme samples fractionated from the culture supernatants were subjected to SDS-PAGE and Western blotting using anti-*Bc*-SMase, -PCPLC, and -PIPLC antibodies. As shown in Fig. 2A, large amounts (>5 µg/ml) of *Bc*-SMase, PCPLC, and PIPLC were detected in the culture supernatants of the clinical isolates, but very small amounts or undetectable levels in those of the ATCC strains. These phospholipase C genes were detected in every clinical and ATCC strain (Figure S1).

Next, we focused on the promoter sequence for the *Bc*-SMase gene (*smase*) or PLC gene (*plc*) of clinical isolates and ATCC isolates. The −35 and −10 promoter sequences of *smase* or *plc* from clinical isolates were almost the same as those of ATCC strains (Fig. 2B and 2C). In *B. cereus,* the transcriptional regulator PlcR (Phospholipase C regulator) controls most known virulence factors [25], [26], and activates gene expression by binding to a nucleotidic sequence called the ‘PlcR box’ [25]. As shown in Fig. 2B and 2C, there was no clear difference in the sequence of the PlcR box between clinical isolates and ATCC strains. In addition, the amino acid sequence of *Bc*-SMase was highly conserved in all strains (Figure S2).

### Effect of Anti-phospholipases on Growth of *B. cereus* in Mice

**Figure 3:**
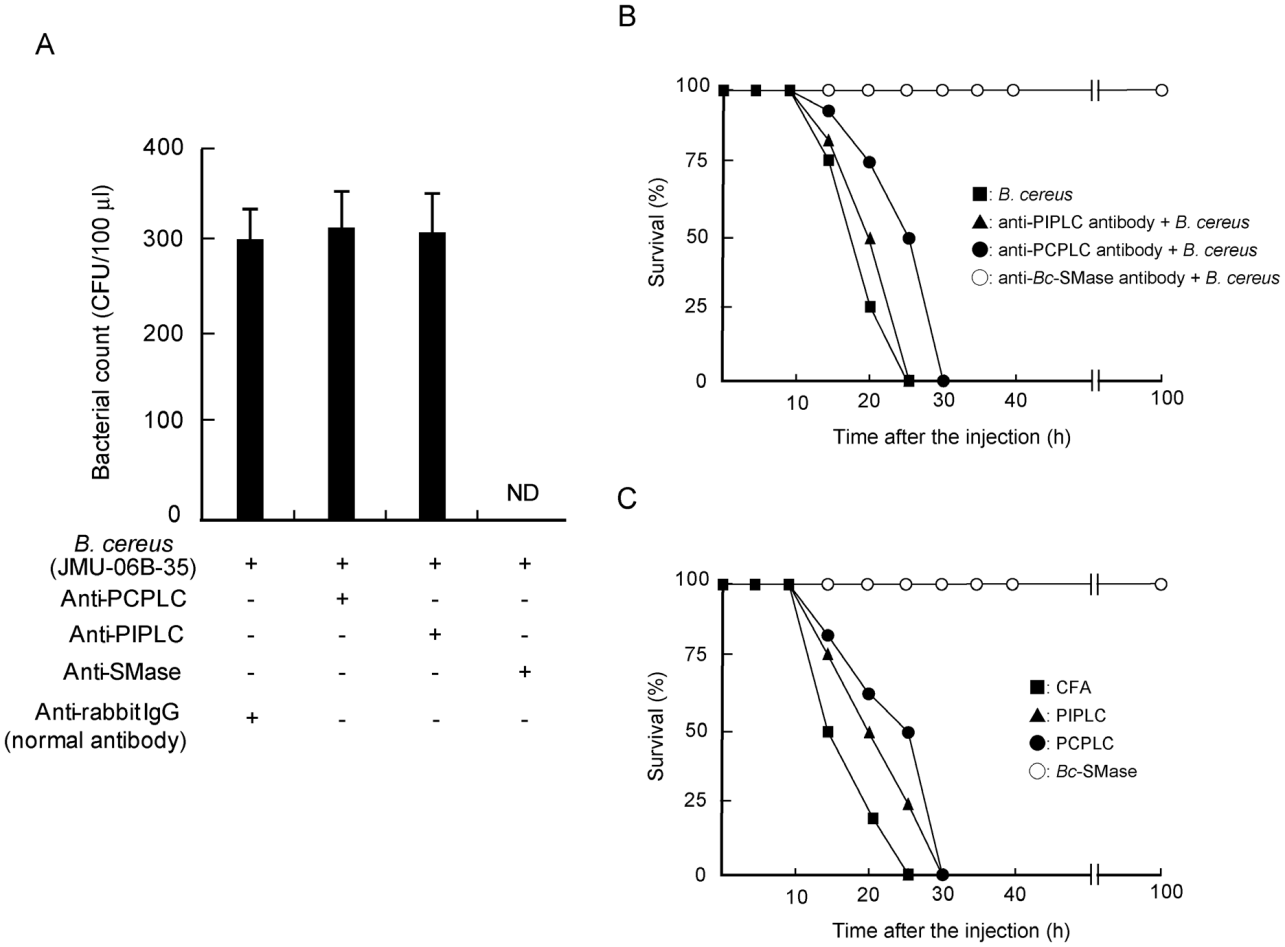
Effect of antibody and immunization against *Bc*-SMase, PCPLC, or PIPLC on lethality of *B. cereus.* Mice intraperitoneally received 50 µg of anti-SMase, -PCPLC, or -PIPLC antibodies and 2 h after the injection, were intraperitoneally administered *B. cereus* (JMU-06B-35). A) *B. cereus* in blood was cultured on Luria Broth agar plates 12 h after the intraperitoneal injection. Values represent the mean ± SEM; *n* = 3 independent experiments. ND: not detected. B) Mice were monitored every five hours after the injection of *B. cereus*. The duration of the experiment was set at 100 h. ▪, *B. cereus*; ▴, anti-PIPLC antibody + *B. cereus*; •, anti-PCPLC antibody + *B. cereus*; ○, anti-*Bc*-SMase antibody + *B. cereus*. C) Mice subcutaneously received an emulsion of the enzyme (*Bc*-SMase (○), PCPLC (•), or PIPLC (▴)) and CFA (▪) 2 times every 2 weeks. The immunized mice received *B. cereus* (JMU-06B-35, 3×10^8^ CFU/mouse). The duration of the experiment was set at 100 h.

To provide clues regarding the growth of *B. cereus* in vivo, the effect of anti-phospholipases on the growth of JMU-06B-35 in mice was investigated. Mice were intraperitoneally injected with the clinical isolate (JMU-06B-35, 5×10^8^ CFU) 2 h after the intraperitoneally administration of 50 µg of anti-PCPLC, -PIPLC, or -SMase antibody. The anti-*Bc*-SMase antibody completely inhibited the growth of JMU-06B-35 in the bloodstream (Fig. 3A). In addition, the mice injected with the anti-*Bc*-SMase antibody did not die within 100 h (Fig. 3B). The administration of the anti-PIPLC and -PCPLC antibodies had no effect on the growth and lethality of JMU-06B-35 in mice (Fig. 3A and 3B). The concentration of these antibodies was enough to neutralize the activity of the three enzymes (10 µg) in vitro (data not shown). It therefore appears that *Bc*-SMase plays an important role in the propagation of *B. cereus* in vivo in our experimental condition.

To confirm the relationship between *Bc*-SMase and the growth of *B. cereus* in vivo, we investigated the effect of immunization of mice with *Bc*-SMase, PCPLC, or PIPLC on the death induced by JMU-06B-35. The BALB/c mice were immunized with 25 µg mixture of PCPLC, PIPLC, or *Bc*-SMase with Complete Freund’s adjuvant (CFA) two times at two-week intervals. Sham-immunized mice administered the clinical isolate began to die after approximately 10 h, and all mice died within 30 h of the administration (Fig. 3C). The survival rate of mice immunized against *Bc*-SMase, PCPLC, or PIPLC was 100%, 0%, and 0% 30 h after infection, respectively (Fig. 3C).

### Effect of *Bc*-SMase on Infection of Mice with ATCC Strain Isolated from Soil

**Figure 4:**
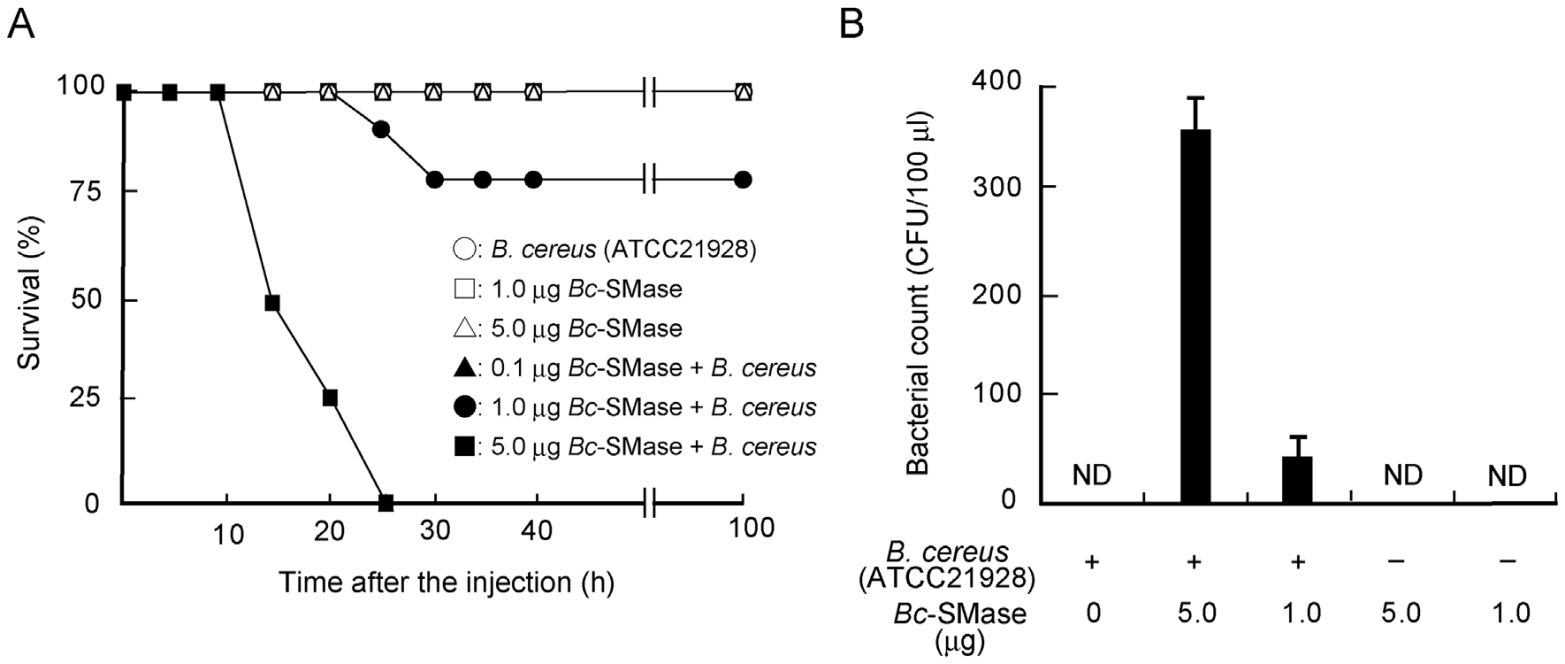
Effect of *Bc*-SMase on the infection with *B. cereus* or *B. subtilis.* Mice received various concentrations of *Bc*-SMase and *B. cereus* (ATCC21928, 5×10^7^ CFU/mouse). A) Mice were monitored every five hours after the injection. The duration of the experiment was set at 100 h. ○, *B. cereus*; □, 1.0 µg *Bc*-SMase; △, 5.0 µg *Bc*-SMase; ▴, 0.1 µg *Bc*-SMase + *B. cereus*; •, 1.0 µg *Bc*-SMase + *B. cereus*; ▪, 5.0 µg *Bc*-SMase + *B. cereus*. B) *B. cereus* in blood was cultured on Luria broth agar plates. Values represent the mean ± SEM; *n* = 5 independent experiments.

To investigate the role of *Bc*-SMase in *B. cereus* infections, we examined the effect of *Bc*-SMase on *B. cereus*-induced death in mice. The animals were intraperitoneally injected with mixtures of ATCC21928 (5.0×10^7 ^CFU/mouse), which did not produce *Bc*-SMase in the culture supernatants, and various concentrations of *Bc*-SMase. As shown in Fig. 4A, the increase in the rate of death was dependent on the dose of *Bc*-SMase above 1.0 µg/mouse. On administration of ATCC21928 and 5.0 µg of *Bc*-SMase, the death rate was 100% within 30 h (Fig. 4A). Mice injected with ATCC21928 or *Bc*-SMase alone survived after 100 h under the experimental conditions (Fig. 4A). In addition, the number of microorganisms in blood 12 h after the administration of the mixture of ATCC21928 and 1.0 or 5.0 µg of *Bc*-SMase was 50–100 and 300–400 CFU/100 µl, respectively (Fig. 4B). On the other hand, the administration of ATCC21928 with PCPLC (5.0 µg/mouse) or PIPLC (5.0 µg/mouse) resulted in no death under the conditions (data not shown).

### Effect of Overexpression of *Bc*-SMase on Growth of *B. cereus or B. subtilis* in Mice

**Figure 5:**
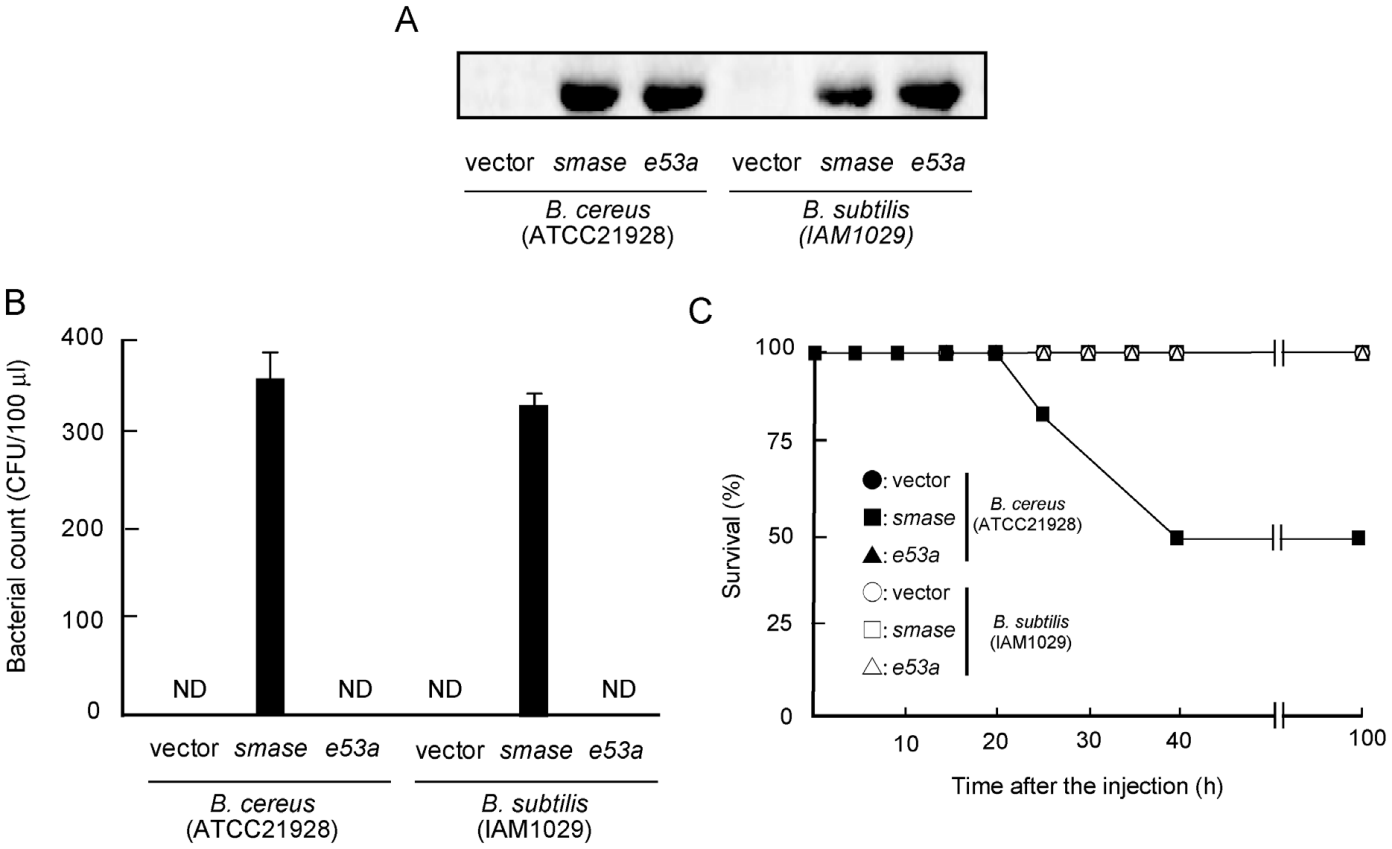
Effect of overexpression of *Bc*-SMase on growth of *B. cereus or B. subtilis* in mice. A) *B. cereus* (ATCC21928) or *B. subtilis* (ISW1215) was transfected with the plasmid carrying *smase* or *e53a*. A) 50% ammonium sulfate precipitation fractions of the culture supernatants (1.0 mg protein) of each microorganism were subjected to SDS-PAGE and Western blotting using anti-*Bc*-SMase antibody. A representative result from one of three experiments is shown. B, C) Mice intraperitoneally received *B. cereus* or *B. subtilis* transformants (1×10^8^ CFU/mouse) carrying empty vector (vector), *smase*, or *e53a*. The microorganisms in the blood of mice about 12 h after the administration of these strains were cultured on Luria Broth agar plates. Values represent the mean ± SEM; *n* = 5 independent experiments. C) Mice were monitored every five hours after the injection. The duration of the experiment was set at 100 h. •, vector (*B. cereus*); ▪, *smase* (*B. cereus*); ▴, *e53a* (*B.cereus*); ○, vector (*B. subtilis*); □, *smase* (*B. subtilis*); △, *e53a* (*B. subtilis*).

To investigate the effect of *Bc*-SMase on growth of *B. cereus* in vivo, we transfected a vector expressing *smase* or the gene for E53A (*e53a*), a variant which has little enzymatic activity [27] (Table S1), into ATCC21928 or *Bacillus subtilis* (ISW1215), which did not produce *Bc*-SMase in the culture supernatants. The ammonium sulfate precipitation fraction of the culture supernatant fluids of these transfected strains was subjected to SDS-PAGE and Western blotting using anti-*Bc*-SMase antibody. As shown in Fig. 5A, these proteins (>5.0 µg/ml) were detected in the culture supernatants of these transformants carrying *smase or e53a*, but not in each microorganism transformed with empty vector. When the mice intraperitoneally received ATCC21928 or ISW1215 transformants carrying the *smase*, the microorganisms were detected about 300–400 CFU/100 µl in bloodstream (Fig. 5B). However, administration of these bacteria carrying *e53a* had no effect on the growth of each microorganism in vivo (Fig. 5B). The results showed that overexpression of *Bc*-SMase in ATCC21928 or ISW1215 induced growth of these strains in vivo. In addition, the survival rate 100 h after administration of ATCC21928 transformants carrying the *smase* was approximately 50%, but that of ISW1215 was 100% (Fig. 5C).

### Effect of *Bc*-SMase on Activation of Macrophages by Peptidoglycan

**Figure 6:**
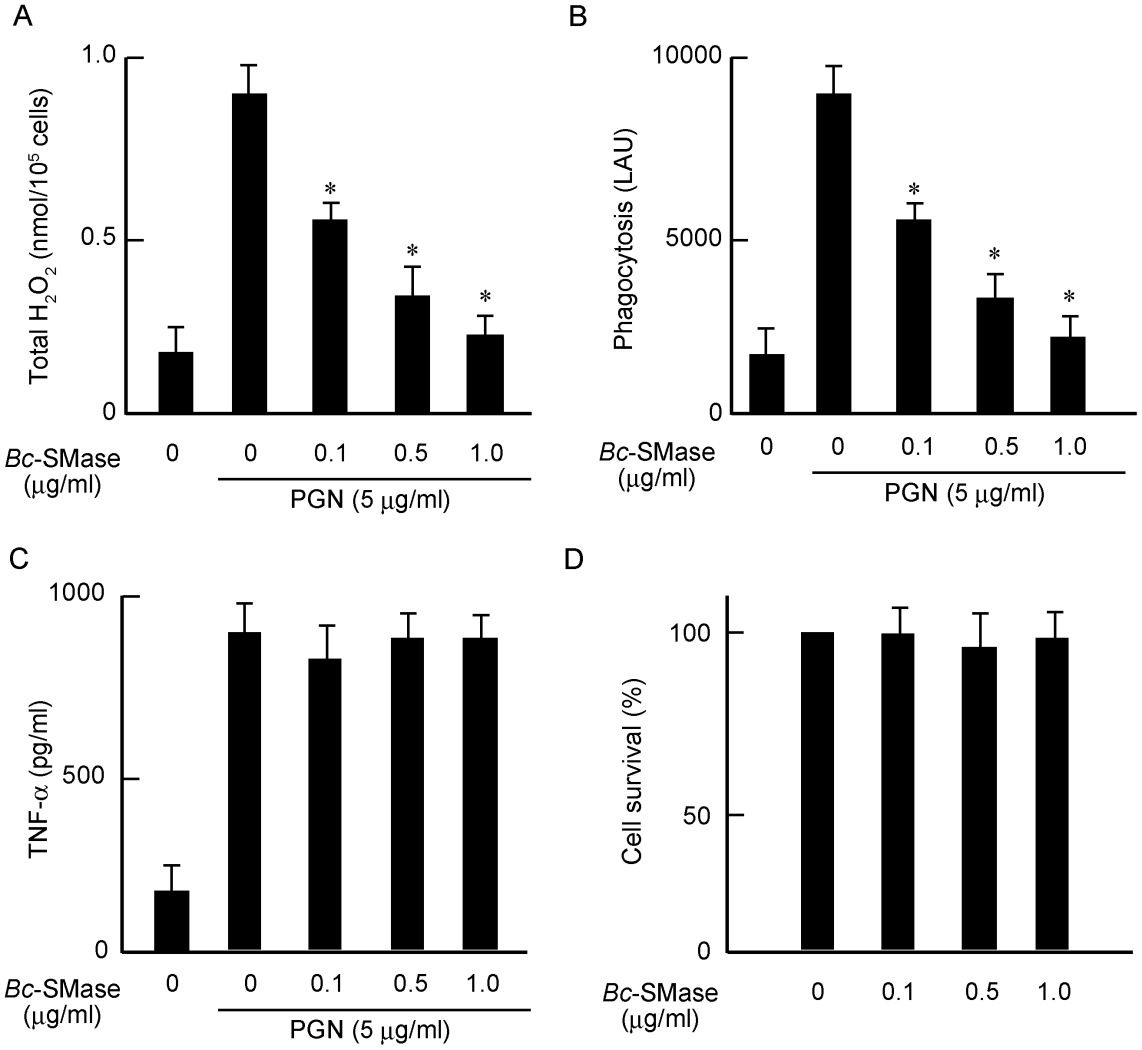
Effect of *Bc*-SMase on activation of mouse macrophages. Mouse macrophages were incubated with or without *Bc*-SMase at 37°C for 60 min (D), and then treated with PGN (5 µg/ml) for 60 min (A, B, C). H_2_O_2_ production, phagocytosis, TNF-α release, and LDH release were measured as described in Materials and Methods. Values represent the mean ± SEM; *n* = 7; * *P*<0.01 compared with H_2_O_2_ production or phagocytosis induced by PGN alone.

González Zorn et al. reported that the SMase from *Listeria ivanovii* mediates bacterial escape from phagocytic cells [15]. The activation of macrophages is known to be related to bactericidal action in vivo. To investigate the effect of *Bc*-SMase on the activation of macrophages, we assessed the effect of PGN, an activator of macrophages, on macrophages treated with *Bc*-SMase. *Bc*-SMase attenuated PGN-activated H_2_O_2_ generation and phagocytosis of macrophages in a dose-dependent manner (Fig. 6A and 6B). However, *Bc*-SMase had no effect on the release of TNF-α induced by PGN from macrophages and induced no release of lactate dehydrogenase (LDH) from the cells (Fig. 6C and 6D). It therefore is likely that *Bc*-SMase specifically influences H_2_O_2_ generation and phagocytosis without impairing membranes of macrophages, suggesting that treatment of macrophages with *Bc*-SMase results in a change in function of the membranes. It was thought that the frustrated phagocytosis may be dependent on the formation of ceramide in macrophage membranes.

### Localization of Ceramide in Membranes of Macrophages Treated with *Bc*-SMase

**Figure 7:**
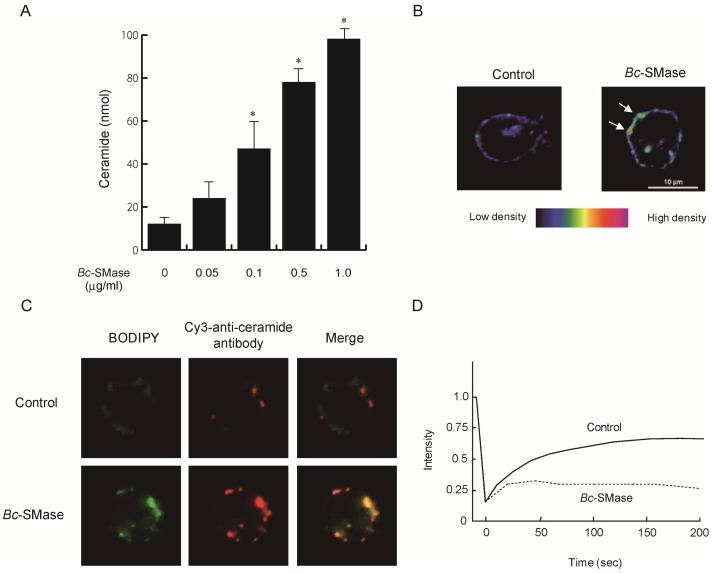
Effect of ceramide on activation of mouse macrophages. A) Mouse macrophages were incubated with various concentrations of *Bc*-SMase at 37°C for 60 min. Ceramide was phosphorylated by 1, 2-diacylglycerol kinase and separated by reverse-phase TLC. Values represent the mean ± SEM; *n* = 3; * *P*<0.01 compared with *Bc*-SMase-untreated cells. (B) Macrophages pretreated with BODIPY-SM were incubated without (left) or with (right) *Bc*-SMase at 37°C for 60 min. (C) Mouse macrophages pretreated with BODIPY-SM were incubated with or without *Bc*-SMase at 37°C for 60 min. The cells were treated with paraformaldehyde, and stained with Cy3-coupled anti-ceramide anti-bodies. (D) Representative recovery curves for the diffusion of BODIPY fluorescence following *Bc*-SMase treatment (broken line, ceramide-rich domain) or without treatment (solid line, ceramide-poor domain) are shown. A representative result from one of ten experiments is shown.

To determine the amount of ceramide formed in the macrophages treated with *Bc*-SMase, macrophages were incubated with *Bc*-SMase at 37°C for 30 min. The lipids extracted from the treated cells were phosphorylated by diacylglycerol kinase from *Escherichia coli,* and developed by reverse-phase thin layer chromatography (TLC). The level of ceramide in the cells treated with *Bc*-SMase increased in a dose-dependent manner (Fig. 7A). Using confocal microscopy, Montes et al. found that phospholipase C from *P. aeruginosa* caused the formation of ceramide-rich domains in biological membranes [28]. We reported that *Bc*-SMase induced the formation of ceramide-rich domains in membranes of sheep erythrocytes and a decrease in the fluidity of membranes, leading to destabilization under physical stimulation [19]. We investigated whether treatment of macrophages with *Bc*-SMase results in the local accumulation of BODIPY-ceramide formed in membranes of cells preincubated with BODIPY FL-C_12_-SM (BODIPY-SM). Fig. 7B (left) shows that the fluorescent substance in membranes of macrophages preincubated with BODIPY-SM was not localized. However, when BODIPY-SM -incubated macrophages were treated with *Bc*-SMase, the local accumulation of the fluorescent substance was found on membranes of the cells, as shown by the white arrows (Fig. 7B, right). To test whether the site where the substance accumulates coincides with a ceramide-rich site, BODIPY–SM-preincubated membranes treated with *Bc*-SMase were analyzed using Cy3-labeled anti-ceramide antibody. As shown in Fig. 7C, the distribution of the fluorescence of the antibody was different from that of BODIPY–SM in the untreated cells. In the case of BODIPY–SM-preincubated macrophages treated with *Bc*-SMase, the location of the fluorescence of Cy3-anti-ceramide antibody was consistent with that of BODIPY, as shown in Fig. 7B, suggesting that BODIPY-ceramide formed from BODIPY-SM in the macrophages treated with *Bc*-SMase is mostly located in ceramide-rich domains. Klein et al. reported that membrane fluidity of cells was evaluated by measurement of lateral diffusion of fluorescence-labeled SM by FRAP with a confocal laser microscopy [29]. A FRAP analysis revealed that the recovery of effective diffusion for the fluorescence of BODIPY in ceramide-rich domains of the macrophages treated with *Bc*-SMase decreased to about 70–80%, compared with that of BODIPY-SM in the untreated cells (Fig. 7D). It therefore appears that the *Bc*-SMase-induced formation of ceramide from SM in membranes of macrophages results in a decrease in the fluidity of membranes.

## Discussion

The present study showed that clinical isolates of *B. cereus,* which produced large amounts of *Bc*-SMase, from patients with sepsis and endophthalmitis were lethal to mice. The production of *Bc*-SMase from clinical isolates was greater than that from ATCC strains under our culture conditions. *B. cereus* produces several secreted toxins, the expression of which is controlled by the PlcR [25], [26]. A difference in protein level from PlcR regulated proteins has been observed for pathogenic factors such as nonhemolytic enterotoxin and hemolysin BL [30], [31]. In addition, variations of the InhA1, NprA, and HlyII, which regulation is independent on PlcR, between pathogenic and nonpathogenic *B. cereus* strains has also been observed [32]. In this study, the sequences of the PlcR box in clinical isolates were almost the same as that in ATCC strains. Therefore, it appears that various factors participate in the pathogenic expression of *B. cereus* and production of *Bc-*SMase.

*Bc*-SMase enhanced the growth of *B. cereus* in the peritoneal cavity, and in addition, invaded the bloodstream in mice, causing death. Furthermore, overexpression of *Bc*-SMase in *B. subtilis*, an avirulent strain, induced growth in mice. The administration of a mixture of ATCC21928 and *Bc*-SMase resulted in the death of mice, but that of PIPLC or PCPLC did not. In addition, the loss of PlcR-regulated factors, which include *Bc*-SMase, significantly attenuated the severity of *Bacillus* endophthalmitis [10]. Furthermore, mice administered the anti-*Bc*-SMase antibody or immunized with *Bc*-SMase were protected from the lethality of clinical isolates of *B. cereus.* Callegan et al., reported that intraocular infection with wild type *B. cereus* or isogenic mutants specifically deficient in PIPLC or PCPLC resulted in similar degrees of destruction of the retinal architecture, and a complete loss of retinal function [33], [34]. These results suggested that *Bc*-SMase plays an important role in the propagation of *B. cereus* in vivo.

Antibody and immunization against *Bc*-SMase protected mice from *B. cereus,* suggesting *Bc*-SMase to be a candidate vaccine against infectious diseases caused by *B. cereus.* Furthermore, it has been reported that nSMase is secreted by several bacteria involved in infectious diseases [17], [18]. Drobnik et al. found that an increase in ceramide levels mediated by nSMase in serum was associated with sepsis-related mortality [35]. Gonzalez-Zorn et al. reported that SMase from *Listeria ivanovii* was involved in avoiding phagocytic vacuoles in the bovine epithelial-cell line MDBK [15]. Heffernan et al. showed that the deletion of three phospholipase (PIPLC, PCPLC, and SMase) genes was required for the attenuation of virulence in a murine model of anthrax and that these enzymes play an important role in the growth of *Bacillus anthracis* in alveolar macrophages [36]. Lo et al. reported that a combination of β-hemolysin immunization and Christie Atkins Munch-Peterson factor neutralization cooperatively suppressed the skin lesions caused by a coinfection of *Staphylococcus aureus* and *Propionibacterium acnes* [37]. The result suggested the need for immunotherapy targeting the interaction of *Staphylococcus aureus* with a skin commensal. Therefore, our findings may provide significant opportunities for the development of new vaccines against infections caused by SMase-producing bacteria.

Treatment of macrophages with SMase resulted in a reduction in the generation of H_2_O_2_ and phagocytosis of macrophages induced by PGN, but no effect on the release of TNF-α and little release of LDH under our experimental conditions. Therefore, it appears that *Bc*-SMase specifically inhibits the activation of macrophages induced by PGN without harmful effects on the cells. The treatment with *Bc*-SMase is known to induce changes in the lipid content of plasma membranes by generating ceramide upon the cleavage of SM [38]. Ceramide is reported to promote the coalescence of rafts, which have been termed “membrane platforms” [39]. We reported that *Bc*-SMase induced the formation of ceramide-rich domains in membranes of sheep erythrocytes, and a decrease in the fluidity of membranes, leading to destabilization under physical stimulation and finally the disruption of erythrocytes [19]. The treatment of mouse macrophages with *Bc*-SMase caused the formation of ceramide, and inhibition of the production of H_2_O_2_ and phagocytosis induced by PGN. Treatment of macrophages with ceramide significantly inhibited the generation of H_2_O_2_ and phagocytosis of macrophages activated by PGN, suggesting that the formation of ceramide induced by *Bc*-SMase is closely related to a reduction in the activation of macrophages. Nakabo and Pabst have reported that C2- or C6-ceramide inhibited the release of superoxides from monocytes primed with lipopolysaccharide, and induced the secretion of small amounts of TNF-α and IL-1β [40]. The treatment of macrophages with *Bc*-SMase resulted in the clustering of ceramide recognized by a Cy3-anti-ceramide antibody. Furthermore, using a confocal laser scanning microscope, we found that the effective diffusion rate for BODIPY-ceramide at ceramide-rich domains in macrophages treated with *Bc*-SMase was reduced about 70%, compared with that for BODIPY-SM, ceramide-poor domains, in control cells. Goni and Alonso reported that the ceramide-rich domains formed a rigid phase in membranes [28], [41]. Therefore, it appears that the formation of ceramide in macrophages treated with *Bc*-SMase results in functional differences of the membranes through the coalescence of ceramide-rich domains and formation of the interface between rigid and fluid domains, leading specifically to inhibition of the production of H_2_O_2_ and phagocytosis induced by PGN.

In conclusion, the hydrolysis of SM to form ceramide in the macrophage membrane treated with *Bc*-SMase induced the attenuation of membrane fluidity and the frustrated phagocytosis. *Bc*-SMase plays a crucial role in the evasion from immune response by macrophages during the early stages of infections of *B. cereus*.

## Materials and Methods

### Strains

The clinical isolates of *B. cereus* (JMU-06B-31, JMU-06B-35, and JMU-06B-1) were isolated at Jichi Medical University. These isolates, obtained from patients diagnosed with *Bacillus* bacteremia or endophthalmitis according to the CDC definition, were identified and characterized as *B. cereus*, as described previously [6]. The ATCC strains of *B. cereus* from soil (ATCC21928, ATCC31429, ATCC6464) were purchased from DS Pharma Biomedical, Tokyo, Japan. The characteristics of the clinical isolates were reported previously [6].

### Mice

Six- to eight-week old male wild-type mice of the ICR and BALB/c strains (Nihon SLC, Japan) were used. Experimental protocols were approved by the Institute Animal Care and Use Committee at Tokushima Bunri University. The mice were housed in plastic cage under controlled environmental conditions (temperature 22°C, humidity 55%). Food and water were freely available.

### Detection of Genes Encoding *Bc*-SMase, PCPLC, and PIPLC

The genomic DNA from various strains of *B. cereus* was extracted with the bacteria genomicPrep Mini Spin kit from GE healthcare (UK). The phospholipase C genes of the genomic DNA were confirmed by PCR using the primer sets described below. *Bc*-SMase primers were forward, 5′-CAAATGGCCAATCGCTGAA-3′, reverse, 5′-GGTTCCTACGTACAGATGCTGGTG-3′. PCPLC primers were forward, 5′-CTTTACAAAGCGTTGCATTTGCTC-3′, reverse, 5′-CAATCGCACGGTTTACAATCCATA-3′. PIPLC primers were forward, 5′-ACCTGATAGTATCCCGTTAGCACGA-3′, reverse, 5′-CGAGCTCCATGGTCCATTTG-3′.

### DNA Cloning and Sequencing

The *plc-smase* region from *B. cereus* (JMU-06B-31, JMU-06B-35, and JMU-06B-1, ATCC21928, ATCC31429, ATCC6464) was obtained as a 2.1-kb DNA fragment by PCR using primer sets described below. A1 forward primer: 5′-GTATTCATTCATTATTCACTGTG-3′, A2 reverse primer: 5′-CTACTTCATAGAAATAGTCGCCT-3′. The fragment isolated from agarose gels was cloned into the vector pGEM-T (Promega, USA). Nucleotide sequencing of the cloned fragments was performed by the dideoxy chain termination technique with a BigDye terminator v1.1 cycle sequencing kit (Applied Biosystems, USA) using M13 reverse and forward primers. The genetic sequence was confirmed with an ABI3500 genetic analyzer (Applied Biosystems, USA).

### Site-directed Mutagenesis

The transformer site-directed mutagenesis kit (LA PCR in vitro Mutagenesis Kit, Takara, Japan) was used with the primer E53A: 5′-GTTATTTTAAATGCCGTGTTTGATAATAGC-3′ to prepare the modified plasmid. The genetic sequence of *Bc*-SMase in each plasmid was confirmed with an ABI310 PRISM™ genetic analyzer (Life technologies, USA).

### Preparation of *Bc*-SMase and Variants

*Bc-*SMase and E53A were overexpressed in *B. subtilis* ISW1214 or *B. cereus* ATCC21928 transformed with the plasmid vector pHY300PLK carrying *smase* or *e53a*. The expression and purification of the recombinant *Bc*-SMase and variants were performed as described previously [21].

### Purification of PCPLC and PIPLC

PCPLC and PIPLC were overexpressed in *B. subtilis* ISW1214 transformed with pHY300PLK carrying the gene of PCPLC or PIPLC cloned from *B. cereus* IAM1029. The PCPLC and PIPLC were secreted into the culture medium. The 80% (w/v) ammonium sulfate fraction of the Luria Broth was fractionated by chromatography using a Cu^2+^-column and then a DEAE-Sepharose column. The purity of samples was verified using SDS-PAGE, and staining with coomasie Brilliant Blue. The PCPLC and PIPLC were observed as a single band at 28 kDa and 34 kDa, respectively.

### Preparation of Antibodies

Anti-*Bc*-SMase, -PCPLC, and -PIPLC antibodies were prepared by immunizing rabbits with 100 µg of the purified phospholipases. CFA (Difco, USA) (1.0 ml) was used for the preparation of antigens. Two hypodermic booster injections were made. Antiserum was obtained 2 weeks after the last injection. Purification of these antisera was performed using Ab-Rapid PuRe (Protenova, Japan).

### Immunization of Mice

Inbred 6–8-week old male BALB/c mice were immunized subcutaneously two times at two-week intervals. The immunogens were used 25 µg mixture of PCPLC, PIPLC, or *Bc*-SMase with CFA.

### ELISA Procedure

The purified recombinant PCPLC, PIPLC, or *Bc*-SMase was diluted to 5 µg/ml in a carbonate buffer (0.05, pH 9.5) and used to coat the wells of polystyrene plates (100 µl/well: Nunc-Immuno plates with a Maxisorp surface) The plates were incubated overnight at 4°C, and the next morning washed three times with PBST (PBS/0.05% Tween-20). The remaining sites of absorption were blocked by the addition of 200 µl/well in PBS containing 3% BSA for 2 h at 37°C. The plates were washed three times with PBST. Serum from each group of immunized animals was serially diluted 2-fold (1∶500 to 1∶128,000) and examined in triplicate wells (100 µl/well) of the blocked antigen-coated plates and incubated for 1 h at 37°C. The plates were then washed five times with PBST and further incubated at 37°C for 1 h with HRP-conjugated anti-mouse IgG (1∶2000). The plates were washed five times with PBST and developed with 100 µl of ortho-pheylenediamine (0.4 mg/ml) in a fleshly prepared citrate phosphate buffer (0.1 M, pH5.0) and H_2_O_2_ (0.4 µg/ml). The reaction was terminated by the addition of 50 µl of 2.5 N H_2_SO_4_/well. Absorbance was read at 492 nm with a microtiter plate reader.

### Determination of ELISA Titer by Endpoint Dilution

The serum was diluted 2-fold from 1∶500 to 1∶128,000, and an absorbance value was determined for each dilution. The cut-off value for the assay was calculated from the reference curve for the control serum. The titer of immune serum was calculated as the reciprocal of the highest dilution yielding a specific optical density above the cut-off value. A significant (*P*<0.05) value of IgG antibody against recombinant PCPLC, PIPLC, and *Bc*-SMase was 128,000, 64,000, and 64,000, respectively, when compared with CFA-treated mouse serum.

### Measurement of Cytokines

The concentration of TNF-α was determined with enzyme-linked absorbent assay kits (R&D systems, USA).

### Culture of Macrophages

Mouse macrophages were isolated from cells in peritoneal exudates with 2 ml of phenol red-free RPMI1640 medium (Wako Pure Chemical Industries, Japan) supplemented with 5% fetal bovine serum (FBS) (Biowest, USA). After centrifugation at 170×g for 10 min at 4°C, the cell pellet was resuspended in phenol red-free RPMI1640 medium supplemented with 5% FBS. Adherent macrophage monolayers were obtained by plating the cells in 96- or 48-well plastic trays (Falcon, USA).

#### Preparation of sheep erythrocytes

Sheep erythrocytes were suspended in 0.02 M Tris-HCl buffer (pH 7.5) containing 0.9% NaCl (TBS), and centrifuged at 1,100×*g* for 3 min. The erythrocytes were washed by the centrifugation three times. The number of erythrocytes was determined with a cell counter (Celltac; Nihon Kohden, Japan).

### Determination of Hemolytic Activity

*Bc*-SMase and E53A was incubated with sheep erythrocytes (12×10^10^ cells/ml) in TBS at 37°C for 30 min, and the cells were chilled at 4°C. The hemolysis of the erythrocytes was measured, as described previously [42]. Hemolysis was expressed as a percentage of the amount of hemoglobin released from 0.1 ml of erythrocytes suspended in 0.4 ml of 0.4% NaCl.

### Preparation of Liposomes

SM (Nacalai Tesque, Japan) from bovine brain and cholesterol (1∶1) in chloroform-methanol (2∶1 v/v) were dried with N_2_ gas, resuspended in TBS containing 0.1 M calboxyfluoroscein (CF). The liposome suspensions were centrifuged at 22,000×g for 15 min at 4°C to remove the nonencapsulated marker, and washed three times by centrifugation. The resulting liposomes were suspended in 200 µl of TBS.

#### The SM-liposome-disruption activity

The SM-liposome-disruption activity was evaluated at the amount of released-CF in the test aliquot. The SM-liposomes in TBS containing 1 mM MgCl_2_ were incubated with *Bc*-SMase or E53A for 30 min at 37°C. The wavelengths for excitation and measurement were 490 and 530 nm, respectively.

### SMase Activity Assay

SMase activity was measured using an Amplex Red Sphingomyelinase assay kit (Invitogen, USA).

### Measurement of Intracellular H_2_O_2_

Mouse macrophages (80% confluent in 48-well plates) isolated from mouse peritoneal exudates were activated with 5 µg/ml PGN (Sigma, USA) for 60 min in the presence of phenol red-free RPMI1640 medium (supplemented with 5% FBS). H_2_O_2_ was measured in the supernatants using an H_2_O_2_ assay kit (Oxis International, USA).

### Assay of Phagocytosis

Phagocytic activity was determined by measuring the uptake of fluorescent microspheres (Fluoresbrite Carboxylate Microspheres, 1.75 µm in diameter, Polysciences), as described [34]. Mouse macrophages (80% confluent in 48-well plates) were stimulated by PGN in the presence of 5.0×10^5^ fluorescent microspheres per ml. After 3 h incubation, cells were washed, and fluorescent intensity in the cells was determined with a fluorescence imaging analyzer (FLA-1000, Fujifilm, Japan).

### Measurement of LDH

LDH activity was determined with LDH assay kits (Wako Pure Chemical Industries, Japan), according to the manufacturer’s instructions.

### Determination of Ceramide

Mouse macrophages were incubated with various concentrations of *Bc*-SMase at 37°C for 60 min in phenol red-free RPMI1640 medium supplemented with 5% FBS. The isolation and the measurement of ceramide were performed as described previously [42], [43]. The ceramide, which is from bovine brain, used as stimulants or standard was purchased from Sigma, USA.

### Immunofluorescence Staining and Confocal Imaging

Mouse macrophages stained with 2 µM BODIPY-SM were plated on 35-mm glass-bottomed dishes (MatTek, USA). The cells were incubated with *Bc*-SMase in phenol red-free RPMI 1640 medium supplemented with 5% FBS at 37°C for 60 min, and the reaction was stopped by 1.0% paraformaldehyde at room temperature. For antibody labeling, the cells were incubated in 50 mM NH_4_Cl in phosphate- buffered saline (PBS) at room temperature for 10 min. After being washed with PBS, the cells were incubated in PBS containing 4% BSA at room temperature for 60 min, followed by mouse Cy3-labeled-anti-ceramide antibody in PBS for 60 min.

### Fluorescence Microscopy

A confocal fluorescence microscope (A1; Nikon, Japan) was used. The excitation wavelength was 488 nm for BODIPY FL C_12_-SM (Molecular probes, USA). The fluorescence signals were simultaneously collected using NIS-Elements C (Nikon software, Japan) into a channel using bandpass filters of 525/50. The objective used in all experiments was a 60×oil immersion, CFI Plan Apo VC 60×oil/1.40 objective (Nikon). The objective lens was used with a zoom factor of 2. The experiments were performed at room temperature.

### Fluorescence Recovery after Photobleaching

Fluorescence recovery after photobleaching (FRAP), a technology used to measure the lateral mobility of membranes, was performed with a Nikon A1R confocal laser scanning microscope, according to the manual. Mouse macrophages stained with 2 µM BODIPY-SM were plated on 35-mm glass-bottomed dishes (MatTek, USA). The photobleaching was performed in a 1.5 µm, visually uniform region of the cell membranes. Bleaching was performed with 5% laser intensity for a duration of approximately 1 s (10 scans of the laser) to achieve 20% bleaching of the BODIPY fluorescence. After photobleaching, images were acquired 200 times at 1 s intervals.

### Statistics

Results were expressed as the mean ± SEM. *n* equals the sample size. Statistical comparisons were performed using an unpaired *t*-test or one-way analysis of variance (ANOVA) with Bonferroni correction. *p* Values less than 0.05 were considered statistically significant.

## Supporting Information

Figure S1 **Detection of genes encoding** ***Bc*** **-SMase, PCPLC, and PIPLC.** The various strains of *B. cereus* were determined for mRNA of *Bc*-SMase, PCPLC, and PIPLC.
(TIF)

Figure S2 **The amino acid sequence alignment of** ***Bc*** **-SMase from clinical isolates or ATCC strains.** The amino acid sequences of *Bc*-SMase from clinical isolates (JMU-06B-1, 31, 35) or ATCC strains (ATCC21928, 31429, 6464) were aligned by the program T-Coffee. The sequences of signal peptide are indicated in bold type. Gray areas indicate amino acid sequence differences. The amino acid residues participating in the enzymatic activity are shown by black circles.
(TIF)

Table S1 **Biological activities of E53A.** Activity (%) was expressed as the percentage of each activity in the wild-type enzyme. Each value is the mean of five experiments.
(DOCX)

## References

[1] Taylor AJ, Gilbert RJ (1975). Bacillus cereus food poisoning: a provisional serotyping scheme.. J Med Microbiol.

[2] Giannella RA, Brasile L (1979). A hospital food-borne outbreak of diarrhea caused by Bacillus cereus: clinical, epidemiologic, and microbiologic studies.. J Infect Dis.

[3] Stenfors Arnesen LP, Fagerlund A, Granum PE (2008). From soil to gut: Bacillus cereus and its food poisoning toxins.. FEMS Microbiol Rev.

[4] Bottone EJ (2010). Bacillus cereus, a volatile human pathogen.. Clin Microbiol Rev.

[5] Drobniewski FA (1993). Bacillus cereus and related species.. Clin Microbiol Rev 1993 Oct;.

[6] Sasahara T, Hayashi S, Morisawa Y, Sakihama T, Yoshimura A (2011). Bacillus cereus bacteremia outbreak due to contaminated hospital linens.. Eur J Clin Microbiol Infect Dis.

[7] Dohmae S, Okubo T, Higuchi W, Takano T, Isobe H (2008). Bacillus cereus nosocomial infection from reused towels in Japan.. J Hosp Infect.

[8] Gaur AH, Shenep JL (2001). The expanding spectrum of disease caused by Bacillus cereus.. Pediatr Infect Dis J.

[9] Van Der Zwet WC, Parlevliet GA, Savelkoul PH, Stoof J, Kaiser AM (2000). Outbreak of Bacillus cereus infections in a neonatal intensive care unit traced to balloons used in manual ventilation.. J Clin Microbiol.

[10] Callegan MC, Kane ST, Cochran DC, Gilmore MS, Gominet M (2003). Relationship of plcR-regulated factors to Bacillus endophthalmitis virulence.. Infect Immun.

[11] Guillemet E, Cadot C, Tran SL, Guinebretiere MH, Lereclus D (2010). The InhA metalloproteases of Bacillus cereus contribute concomitantly to virulence.. J Bacteriol.

[12] Guinebretiere MH, Broussolle V, Nguyen-The C (2002). Enterotoxigenic profiles of food-poisoning and food-borne Bacillus cereus strains.. J Clin Microbiol.

[13] Ramarao N, Lereclus D (2005). The InhA1 metalloprotease allows spores of the B. cereus group to escape macrophages.. Cell Microbiol.

[14] Tran SL, Guillemet E, Ngo-Camus M, Clybouw C, Puhar A (2011). Haemolysin II is a Bacillus cereus virulence factor that induces apoptosis of macrophages.. Cell Microbiol.

[15] Gonzalez-Zorn B, Dominguez-Bernal G, Suarez M, Ripio MT, Vega Y (1999). The smcL gene of Listeria ivanovii encodes a sphingomyelinase C that mediates bacterial escape from the phagocytic vacuole.. Mol Microbiol.

[16] Tseng HJ, Chan CC, Chan EC (2004). Sphingomyelinase of Helicobacter pylori-induced cytotoxicity in AGS gastric epithelial cells via activation of JNK kinase.. Biochem Biophys Res Commun.

[17] Collins J, Buckling A, Massey RC (2008). Identification of factors contributing to T-cell toxicity of Staphylococcus aureus clinical isolates.. J Clin Microbiol.

[18] Schwan WR, Langhorne MH, Ritchie HD, Stover CK (2003). Loss of hemolysin expression in Staphylococcus aureus agr mutants correlates with selective survival during mixed infections in murine abscesses and wounds.. FEMS Immunol Med Microbiol.

[19] Oda M, Takahashi M, Matsuno T, Uoo K, Nagahama M (2010). Hemolysis induced by Bacillus cereus sphingomyelinase.. Biochim Biophys Acta.

[20] Openshaw AE, Race PR, Monzo HJ, Vazquez-Boland JA, Banfield MJ (2005). Crystal structure of SmcL, a bacterial neutral sphingomyelinase C from Listeria.. J Biol Chem.

[21] Ago H, Oda M, Takahashi M, Tsuge H, Ochi S (2006). Structural basis of the sphingomyelin phosphodiesterase activity in neutral sphingomyelinase from Bacillus cereus.. J Biol Chem.

[22] Huseby M, Shi K, Brown CK, Digre J, Mengistu F (2007). Structure and biological activities of beta toxin from Staphylococcus aureus.. J Bacteriol.

[23] Bryant AE, Chen RY, Nagata Y, Wang Y, Lee CH (2000). Clostridial gas gangrene. I. Cellular and molecular mechanisms of microvascular dysfunction induced by exotoxins of Clostridium perfringens.. J Infect Dis.

[24] Marques MB, Weller PF, Parsonnet J, Ransil BJ, Nicholson-Weller A (1989). Phosphatidylinositol-specific phospholipase C, a possible virulence factor of Staphylococcus aureus.. J Clin Microbiol.

[25] Gohar M, Faegri K, Perchat S, Ravnum S, Okstad OA (2008). The PlcR virulence regulon of Bacillus cereus.. PLoS One.

[26] Lereclus D, Agaisse H, Gominet M, Salamitou S, Sanchis V (1996). Identification of a Bacillus thuringiensis gene that positively regulates transcription of the phosphatidylinositol-specific phospholipase C gene at the onset of the stationary phase.. J Bacteriol.

[27] Obama T, Kan Y, Ikezawa H, Imagawa M, Tsukamoto K (2003). Glu-53 of Bacillus cereus sphingomyelinase acts as an indispensable ligand of Mg2+ essential for catalytic activity.. J Biochem.

[28] Montes LR, Lopez DJ, Sot J, Bagatolli LA, Stonehouse MJ (2008). Ceramide-enriched membrane domains in red blood cells and the mechanism of sphingomyelinase-induced hot-cold hemolysis.. Biochemistry.

[29] Klein C, Pillot T, Chambaz J, Drouet B (2003). Determination of plasma membrane fluidity with a fluorescent analogue of sphingomyelin by FRAP measurement using a standard confocal microscope.. Brain Res Brain Res Protoc.

[30] Duport C, Thomassin S, Bourel G, Schmitt P (2004). Anaerobiosis and low specific growth rates enhance hemolysin BL production by Bacillus cereus F4430/73.. Arch Microbiol.

[31] Fagerlund A, Brillard J, Furst R, Guinebretiere MH, Granum PE (2007). Toxin production in a rare and genetically remote cluster of strains of the Bacillus cereus group.. BMC Microbiol.

[32] Cadot C, Tran SL, Vignaud ML, De Buyser ML, Kolsto AB (2010). InhA1, NprA, and HlyII as candidates for markers to differentiate pathogenic from nonpathogenic Bacillus cereus strains.. J Clin Microbiol.

[33] Callegan MC, Cochran DC, Kane ST, Gilmore MS, Gominet M (2002). Contribution of membrane-damaging toxins to Bacillus endophthalmitis pathogenesis.. Infect Immun.

[34] Callegan MC, Jett BD, Hancock LE, Gilmore MS (1999). Role of hemolysin BL in the pathogenesis of extraintestinal Bacillus cereus infection assessed in an endophthalmitis model.. Infect Immun.

[35] Drobnik W, Liebisch G, Audebert FX, Frohlich D, Gluck T (2003). Plasma ceramide and lysophosphatidylcholine inversely correlate with mortality in sepsis patients.. J Lipid Res.

[36] Heffernan BJ, Thomason B, Herring-Palmer A, Shaughnessy L, McDonald R (2006). Bacillus anthracis phospholipases C facilitate macrophage-associated growth and contribute to virulence in a murine model of inhalation anthrax.. Infect Immun.

[37] Lo CW, Lai YK, Liu YT, Gallo RL, Huang CM (2011). Staphylococcus aureus hijacks a skin commensal to intensify its virulence: immunization targeting beta-hemolysin and CAMP factor.. J Invest Dermatol.

[38] Ikezawa H, Mori M, Ohyabu T, Taguchi R (1978). Studies on sphingomyelinase of Bacillus cereus. I. Purification and properties.. Biochim Biophys Acta.

[39] Gulbins E, Dreschers S, Wilker B, Grassme H (2004). Ceramide, membrane rafts and infections.. J Mol Med.

[40] Nakabo Y, Pabst MJ (1997). C2-ceramide and C6-ceramide inhibited priming for enhanced release of superoxide in monocytes, but had no effect on the killing of leukaemic cells by monocytes.. Immunology.

[41] Goni FM, Alonso A (2009). Effects of ceramide and other simple sphingolipids on membrane lateral structure.. Biochim Biophys Acta.

[42] Ochi S, Oda M, Matsuda H, Ikari S, Sakurai J (2004). Clostridium perfringens alpha-toxin activates the sphingomyelin metabolism system in sheep erythrocytes.. J Biol Chem.

[43] Oda M, Matsuno T, Shiihara R, Ochi S, Yamauchi R (2008). The relationship between the metabolism of sphingomyelin species and the hemolysis of sheep erythrocytes induced by Clostridium perfringens alpha-toxin.. J Lipid Res.

[44] Notredame C, Higgins DG, Heringa J (2000). T-Coffee: A novel method for fast and accurate multiple sequence alignment.. J Mol Biol.

