# Supplementary figures and images for: Role of Sphingomyelinase in Infectious Diseases Caused by Bacillus cereus
Source: PLoS One. 2012 Jun 6;7(6):e38054. doi: 10.1371/journal.pone.0038054 (PMC3368938; doi:10.1371/journal.pone.0038054)

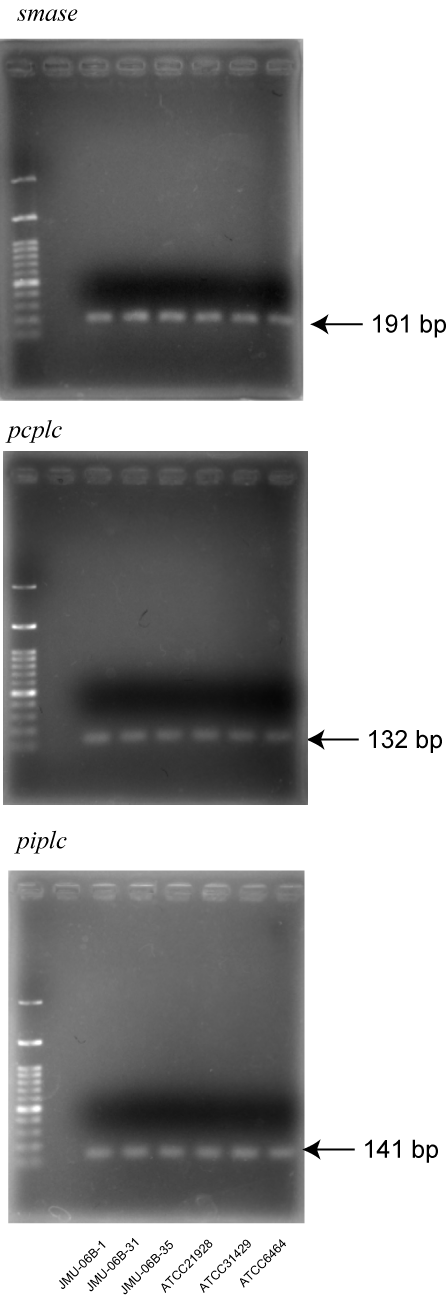

Supplement: Figure S1 — Detection of genes encoding Bc -SMase, PCPLC, and PIPLC. The various strains of B. cereus were determined for mRNA of Bc-SMase, PCPLC, and PIPLC. (TIF) [file pone.0038054.s001.tif]

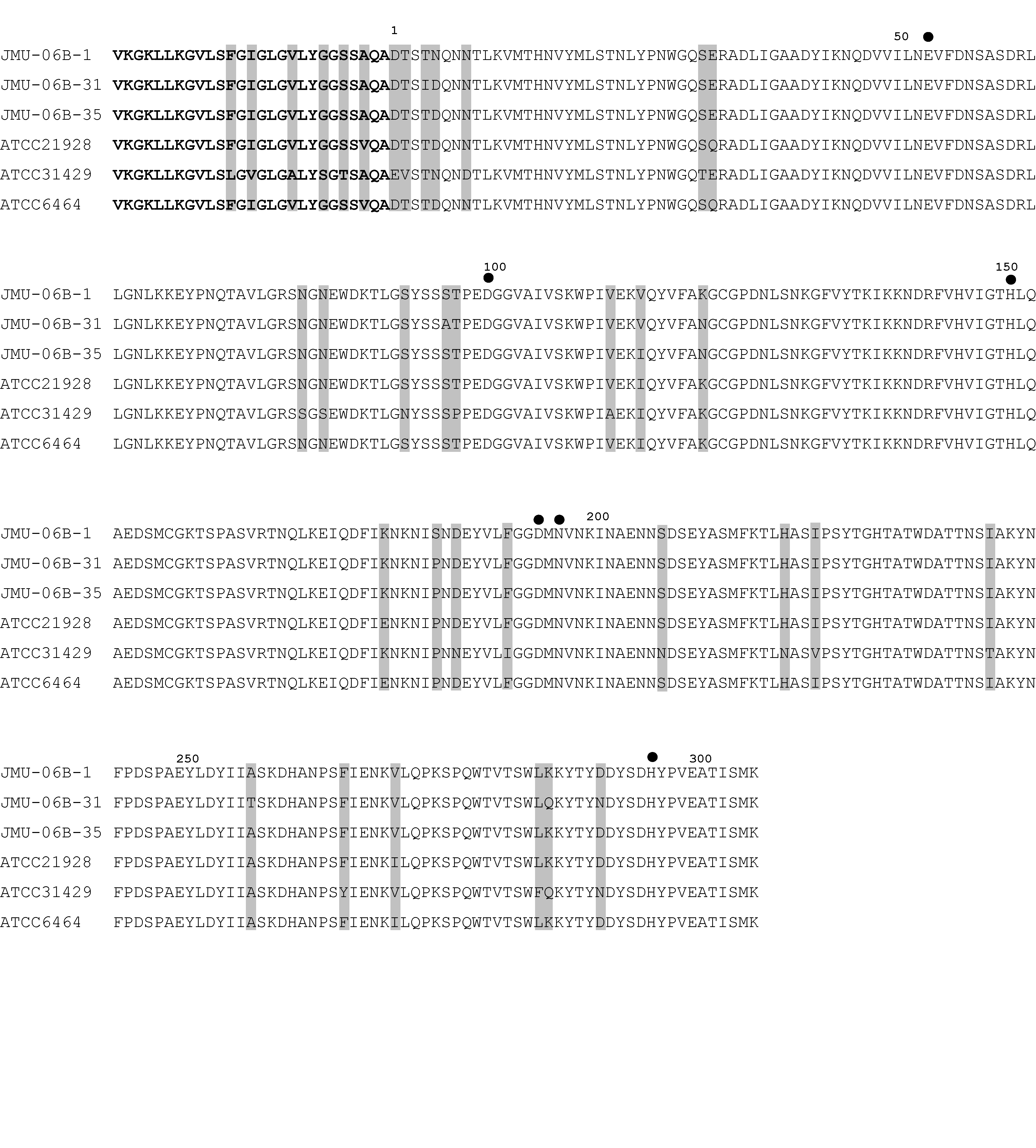

Supplement: Figure S2 — The amino acid sequence alignment of Bc -SMase from clinical isolates or ATCC strains. The amino acid sequences of Bc-SMase from clinical isolates (JMU-06B-1, 31, 35) or ATCC strains (ATCC21928, 31429, 6464) were aligned by the program T-Coffee. The sequences of signal peptide are indicated in bold type. Gray areas indicate amino acid sequence differences. The amino acid residues participating in the enzymatic activity are shown by black circles. (TIF) [file pone.0038054.s002.tif]
