# Supplementary material for: Role of Sphingomyelinase in Infectious Diseases Caused by Bacillus cereus
Source: PLoS One. 2012 Jun 6;7(6):e38054. doi: 10.1371/journal.pone.0038054 (PMC3368938; doi:10.1371/journal.pone.0038054)
Supplement: Table S1 — Biological activities of E53A. Activity (%) was expressed as the percentage of each activity in the wild-type enzyme. Each value is the mean of five experiments. (DOCX) [file pone.0038054.s003.docx]

**Supplemental Table S1**

| **Enzymes** | **Hemolytic activity (%)** | **CF-release from**  **liposomes (%)** | **Sphingomyelinase**  **Activity (%)** |
| --- | --- | --- | --- |
| **Wild-type** | 100 | 100 | 100 |
| **E53A** | > 0.01 | > 0.01 | > 0.01 |
